# Editorial Chit-Chat

**Published:** 1876-01

**Authors:** 


### Editorial Chit-Chat.

FIFTY-FIVE CENTS.—Enclosed in this number, is the yearly envelope, in which to send your remittance for the next volume.

JAMES VICK—The Rochester Florist, has an advertisement in this number of the Bistoury. We recommend all lovers of flowers to send for the Florist's Guide, published by him, and containing much useful information upon the subject of flower growing.

RUSSIAN OVERCOAT.—The most comfortable looking garment for a gentleman, accustomed to riding out in the cold, is worn by a prominent physician of this city. It is an overcoat, long enough to cover head and heels, made of astrakhan fur, and was manufactured in Russia.

ENCYCLOPEDIAS.—We have one—it cost us considerable money. We have had occasion to refer to it six times within a week, and in neither instance could we find what we desired to know.—As the information sought for *should* have been there, we vote cyclopedias in general, and Appleton's in particular—well, not what they should be.

THAT STORY.—We hope that none of our readers will skip the capital story related so graphically by Dr. Kilpatrick of Texas, in this number of the BISTOURY. The Doctor proves to be a capital writer of sketches, and promises another story for the next number. We are sure all will invite him to come again.

Dr. W. C. DOANE, formerly of Williamsport, has removed to Syracuse, where he has fitted up an elegant and elaborate suit of rooms, intended for an office and consultation practice. Dr. Doane has been engaged in a vigorous and laborious practice, for the past twenty-five years, during which time he has become widely known as an unusually successful diagnostician. The Doctor is therefore peculiarly fitted for a consulting physician, and we are glad to hear that his skillfulness and learning has already secured to him a large practice in the city of his adoption.

THE REMINDER.—Our friends will find the envelope and blank in this number of the BISTOURY, reminding them of the close of the year, and of volume eleventh of this journal. We hope all will respond promptly with their *fifty-five cents*, and so renew their subscription for 1876. We expect to be able to devote more time to our journal during the coming year, and so increase its usefulness, rendering it invaluable in every household. We believe there is no publication in existence, afforded at so small a price, that is of such general usefulness, as the BISTOURY. The coming year, will be the twelfth of its life. Send in your subscriptions early, and try add a new name to our subscription list.

A STRONG LETTER.—We have been shown the following letter, written to a gentleman congratulating him upon his recovery from a severe illness. We give it as exceedingly interesting reading:

"My Dear Sir:—I see from the daily Journals—and what they don't know is past finding out,—that you have been ill; happily, now restored to health again. I learn that you had gastrenteromalacia, said to be an exceedingly painful disorder, implicating the gastroepeploric-nerves, and so producing a pain closely resembling nephrolithiasis.—I hope it may not assume a chronic form and become gastroenteritis, for then one may exhaust the entire nosochthouographia in search of an effective remedy. I hope you may escape further attacks from this disease, that your temporomaxilliary articulation may remain intact, your powers of deglutition unimpaired, with no obstruction in your œsophagus; then, with proper cardiac function, you will be able to dispel all tendency to lypothymia, and to digest and assimilate a good dinner as well as all letters of this character.

Yours, &c., ———.

PARK CHURCH FAIR.—The recent Fair at Park Church, in this city, of which Rev. Thomas K. Beecher is pastor, was a well arranged and manipulated affair from beginning to end. The large church edifice is peculiarly adapted to festivals of this nature. The lecture room, the romp room, curiosity shop, and church parlors were crowded with many spectators during the three days of the festival. The attractions were numerous, not the least of which was the wonderful white elephant, whose elephantine tricks amused and delighted both the children and their parents. It was constructed by Mrs. Beecher, and was certainly one of the most ingenious representations of animal life that ever emanated from a "property-room." The color, proportions, and movement of trunk, ears, tail, and limbs, were perfection, and when the animal "took up" the contents of a pail of water in his trunk and spirted it about his head, and "trumpeted" his seeming delight through this same nasal appendage, to the consternation of the smaller spectators, one could scarcely divest himself of the impression that the largest animal of some menagerie had been let loose for the benefit of the occasion. In short, the elephant was a magnificent success, and deserved the three hundred dollars which it earned for the fair fund.

---

QUACK ALMANACS.—Of late years, the almanac manufacturing and dispensing business has been wholly monopolized by patent medicine men. To consult an almanac in order to learn upon what particular day of the week your last baby was born, or when it was your mother-in-law fell down stairs and dislocated her hip, or for any other equally amusing event, you have staring you in the face, an advertisement in which the wonderful curative virtues of some quack medicine is extolled. On every page, with the rising of the sun, will also be indicated the rising of some almost defunct individual, through swallowing Dr. Stickinthemud's pulmonic cough slayer, or Dr. Physickyough's anti-billious pills, sea-weed tonic, Vinegar Bitters, Catarrh Remedies, Sarsaparilla, Cough Drops, Rheumatic Liniments, and the Lord only knows what else,—recommended for every disease known to mortal man, and for many not yet discovered, and probably never *will* be.—Under this condition of affairs, it is extremely wholesome and indicative of a return to the age of reason, that we are now being supplied with an almanac, without a solitary quack medicine advertisement in it! The credit of issuing such a pamphlet, is due to E. Steiger, Publisher, of New York, and is called "The Popular Health Almanac." In lieu of the usual patent medicine literature, is given sound advice, and information how to act in case of drowning, or other accidents, and what to do in sudden and severe illness, until the doctor comes. Antidotes to poisons are given, and the composition of the quack medicines of the day. The pamphlet is a very useful one, and should be found in every family. Bidwell, the druggist, will give you one.

---

COPENHAGEN.—Not the city of that name in Denmark, but the modern institution of our country. While we write, our fifteen-year-old son is celebrating his birth day, with his schoolfellows and misses, in the next apartment. We hear the merry laughter and the jolly rompings of the boys and girls, and as we stroke our gray hairs and twist our beard between thumb and finger, thinking of long ago, a sudden smack, as though a chemist's retort had exploded, greets our ear—then another,—and another still! The sound rings so familiarly and so frequently withal, that wife, obedient to our look of inquiry, opens the door softly, peers into the room, then, smilingly turns to us and remarks, casually, "Copenhagen!"

The smacking continues, in fact the pyrotechnics become alarmingly frequent; another look of nervousness toward the door, eliciting the same reply—"Copenhagen!" Finally, we throw down our pencil, open the door from whence these effervescing ebullitions, when we discover a circle of healthy, rosy cheeked, neatly clad boys and girls, and in the circle, that young hopeful of ours, with his arms about a struggling miss, striving to kiss her! We enquire anxiously, what's up? "Copenhagen," is the only response, and the kissing bee continues, with the old gentleman in the circle to see (mind you) that "the means of refreshment are not perverted to intemperance or excess." Copenhagen!

---

THE SURGICAL INSTITUTE.—Dr. James A. Hall, one of the most accomplished young physicians in this city, has become connected with the Surgical Institute. He has been, for some time, the Secretary of the Elmira Academy of Medicine, and Vice President of the Chemung County Medical Society, which prominent positions in the faculty is a full guaranty of his worth and skill as a physician. We are quite sure that Dr. Up de Graff and his patients will find the presence of Dr. Hall in the Institute something to their advantage and benefit. Dr. Up de Graff, with the great success his Institute has met with, has found that the care of it has considerably overworked him, and he cannot but begin to feel the effects of it upon his health. He could hardly have made a better choice for an assistant than Dr. Hall.—*Daily Advertiser.*

It will be seen from the above, that we have secured very competent help, of which we have long been in need. With Dr. Hall's intelligent assistance, we feel that much of the detail of conducting a large establishment like ours has been removed from our shoulders, giving us much needed rest. It also gives us an opportunity to devote more time to the BISTOURY, which we think our friends will discover in this and subsequent issues of our journal.

---

FEELINGLY.—"Ten to one," as the sporting man has it, that the woman who is constantly grumbling of her hotel fare, has had the plainest of diet at home. When she speaks of her wealthy kindred, and how she misses her horse and carriage, it will be a safe investment to wager that she had but one alternative—to marry or go to work, and that her "turnout" at home will be found to be a cart-horse and truck.

When she expatiates upon the usages of good society, she invariably exhibits her own ignorance of it.

When she inquires into the antecedents of her neighbors, speculating upon their reliability, commenting upon their appearance, and making ill-natured remarks upon their style of dress, it is a certain indication that she herself is ill-bred, lacks taste in ornamentation of person and the means of supplying her wants.

In short, wherever you find a bigoted, ignorant, gossiping, treacherous woman, in any public or private institution, you have discovered the devil incarnate, capable of disorganizing a household, a church, a community or an empire. Confound them! And as Patrick aptly remarks, "may the divil fly away with all sich."

---

COMPLIMENTS OF THE SEASON.—When Richard III gave vent to his feelings in "now is the winter of our discontent," we opine it to have been just before Christmas, when Mrs. Richard had sent him down town in quest of presents for the little Richards, and the balance of the household. Can anything be more perplexing and demoralizing than to enter a modern toy shop, where the shelves, counters and floors are covered with all manner of contrivances to amuse the little folks and harass the older ones? Whistles, trumpets, horns, drums, rattles and multitudinous other noise-creating devices, calculated to make "each particular hair stand on end," when bedlam is let loose on Christmas morning.

There you see myriads of impossibly shaped animals, with squeaking voices and rickety wheels, crooked-legged and cross-eyed. Noah's arks, full of the smaller and more fragile sort, to identify which, would puzzle the ancient ship-builder himself. There, with the jack's-in-the-box, monkeys-on-a-stick, tin carts, tin horses, hoops, bows and arrows, and the mechanical toys, make up a list that is simply bewildering. After looking at, inquiring the price of, and handling everything in the concern, you almost settle upon taking something, but fear it isn't "just the thing"—look at something else—are still in doubt—look again—are perplexed, and while the good natured lady behind the counter, follows you about, suggesting this, that and the other, you become completely and wholly exasperated; when, wiping the perspiration from your brow, you mumble some excuse about calling with your wife, and then rush abstractedly from the toy repository, determined upon delegating to some more tranquil person of the household, all your right and title in the christmas-gift-purchasing-business.

---

CHRISTMAS.—It is almost here, and we are hanging around the edges of it, as it were, partaking more or less of the excitement of the little folks. In fact, it is Christmas eve, and we had congratulated ourself that "copy" was all in for the BISTOURY, and that we had nothing to do but listen to the busy prattle of the children, while they arrange their stockings, suspended from chairs, sofas, tables, and every other eligible point, that Santa Claus might not overlook them. We had settled down, we say, in our big, easy chair, with feet to the glowing grate, when the door-bell tingled with a mighty thrill, which sent the children scampering to their beds, with exclamations on every lip—"Santa Claus!" "Santa Claus has come!" The servant soon returns and announces that the printer is at the door, waiting for copy for the BISTOURY—"says he lacks a page and a half!" Hail Columbia! we exclaim, in sheer despair; will the wants of that printer *never* be satisfied? It seems to us that sufficient copy has already been furnished equal to two numbers of such a journal! But the printer is waiting, and the copy must be forthcoming.

While we write, a snow storm is opportunely hiding the nakedness of the earth, and is covering the evergreen trees with beautiful snowflakes, until their limbs bow gracefully to the weight, making their silent obeisance to the joyful season. For this, we rejoice; for, aside from the sombre, aspect of a "green Christmas," the disappointment of the children, all over this northern clime of ours, at not having snow upon which to try the new, bright sleighs which Santa Claus is sure to bring to many of them, would cause a pang in their little hearts, when all should be merry and happy. Christmas is peculiarly a day for the children, and that it is so, is a matter for congratulation, for we are sure that older hearts rejoice that a little care and forethought as to supplying the wants and desires of the dear ones, should bring them so much happiness. Fill up the stockings, therefore, to-night! Fill them to the very top, and let them run over, even, with buns, candies, toys, and the many little things so dear to the child's heart. And when the morrow dawns, long, long before daylight comes peeping through the blinds, you will hear the gentle patterings of wee feet upon the stairs, and the muffled whisperings of the little rogues, as they steal down, and glide noislessly into the parlor to see what Santa Claus has brought to them during the long and weary night. By-and-by, the silence can no longer be maintained, when a regular outburst of laughter and merriment greets your ear, and by the time the happy little elfs reach your bedside with armsful of trinkets to exhibit to your wondering eyes, you are ready to be up and dressed for the frolic in hand. Happiness! It is only personified in a little child, with an armful of toys, on Christmas morning.

---

A HOOKED ADDER IN A MAN'S STOMACH.—George M. Ball, a young man of eighteen years, employed on the farm of Mr. Berry, Springfield, Mass., and who has had an extremely voracious appetite for some time, has been accustomed to drink, while milking night and morning, a quantity of warm milk. A few mornings since, failing to take his accustomed draught, something came up in his throat, choking him, and he fell senseless. A son of Mr. Berry was surprised to see a snake's head protruding from Ball's mouth, but on attempting to seize it, the serpent retreated down his throat. A powerful emetic was administered and in a short time the young man vomited up a "hooked adder" two feet and eight inches long, and about as thick as two fingers of a man's hand. It lived only five minutes. Ball has probably carried the serpent for at least twelve years, as he was accustomed to drink from a small brook when a boy. Since parting with his tenant his health has greatly improrved and his appetite is a little more reasonable.—*New York Era.*

Now and then, we stumble upon an item like the above, written by some innocent country editor, who is not supposed to be well versed in physiology nor natural science. But when a city editor gives place to such a ridiculous story in his newspaper, he deserves to have a "hooked adder" fastened in his nose, as a perpetual reminder of his ignorance and gullibility. *If* there exists such a young man as Geo. M. Ball, and *if* he really did tell such a story as the above, why, he's a "fraud," or to use more emphasis, in keeping with the requirements of the case,—he's a liar.

Any boy, a graduate of the high school, could tell the *Era* man, that a snake of any species, be he "hooked," ring-tailed or striped, could not exist in the stomach an hour, much less for twelve years! Nor could *any* reptile of any species whatever, so live, for the reason that they breathe by means of lungs, and could no more exist in the stomach than they could in the exhausted receiver of an air pump. Then, could the animal be supplied with air, by any means peculiar to Mr. Ball's anatomy, the case would be no better for the poor snake, for the gastric and other juices of the stomach would dissolve and convert him into soup in less than two hours. For this reason *entozoa*, (adapted to living in closed cavities,) tape-worms and round worms, cannot live in the stomach but are always found in the intestines. When they do wander into the stomach—as sometimes happens—they are quick to find an exit by way of the throat, and creep out at the mouth.

People sometimes *imagine* that living animals exist in their stomachs, to creep out at their mouths, nostrils and ears and annoy them with hissing sounds or their forked tongues. But such folks are monomaniacs.

Several years ago a young man applied to us for relief from a serpent he had swallowed, in minature form, at the traditional brook, "where he had been in the habit of drinking." All arguments to convince him that such an animal could not live in his stomach, were utterly unavailing, for he knew to a certainty that the animal protruded its head from his mouth, and frequently hissed in his ears. He suggested a trap, baited with salt-pork, for its capture, as the animal was very fond of that diet, and troubled him most with its restless attacks, when he had dined upon that luscious morsel. Perceiving the earnestness and determination of the lad, we directed him to call in a week, when we would have something in readiness for the capture of his troublesome enemy. Promptly, upon the day appointed, he made his appearance. In the mean time, we had captured a small "garter-snake," and kept him alive and hearty for our experiment. We gave the young man an emetic, and while his head was held over the receptacle which was receiving the contents of his stomach, and at the moment when the most vigorous gush of dinner came from his mouth, we adroitly dropped the snake into the vessel, upon which the young man exclaimed—"There it is! there it is! I told you so! O, see him creep, that's just the way he behaved in my stomach!" and with many words of thanks for the relief afforded, he took his departure, and ever afterwards remained well.

Perhaps some physician played the same successful experiment upon Mr. Ball. If so, still no excuse remains for the *Era* man's ignorance in the matter.

